# Development and validation of a large animal ovine model for implant-associated spine infection using biofilm based inocula^☆^

**DOI:** 10.1016/j.bioflm.2023.100138

**Published:** 2023-06-27

**Authors:** Jeremy D. Shaw, Travis L. Bailey, Jemi Ong, Darrel S. Brodke, Dustin L. Williams, Richard A. Wawrose, Richard T. Epperson, Brooke Kawaguchi, Nicholas N. Ashton

**Affiliations:** a Department of Orthopaedics, University of Utah, Salt Lake City, UT, USA; b Department of Orthopaedic Surgery, University of Pittsburgh, Pittsburgh, PA, USA; c Department of Pathology, University of Utah, Salt Lake City, UT, USA; d Department of Biomedical Engineering, University of Utah, Salt Lake City, UT, USA; e Department of Physical Medicine and Rehabilitation, Uniformed Services University of the Health Sciences, Bethesda, MD, USA

**Keywords:** Infection, Biofilm, Spondylodiskitis, Epidural, Failed back surgery, Prosthesis, Pedicle screw

## Abstract

Postoperative implant-associated spine infection remains poorly understood. Currently there is no large animal model using biofilm as initial inocula to study this challenging clinical entity. The purpose of the present study was to develop a sheep model for implant-associated spine infection using clinically relevant biofilm inocula and to assess the *in vivo* utility of methylene blue (MB) for visualizing infected tissues and guiding debridement. This 28-day study used five adult female Rambouillet sheep, each with two non-contiguous surgical sites– in the lumbar and thoracic regions– comprising randomized positive and negative infection control sites. A standard mini-open approach to the spine was performed to place sterile pedicle screws and *Staphylococcus aureus* biofilm-covered (positive control), or sterile (negative control) spinal fusion rods. Surgical site bioburden was quantified at the terminal procedure. Negative and positive control sites were stained with MB and staining intensity quantified from photographs. Specimens were analyzed with x-ray, micro-CT and histologically. Inoculation rods contained ∼10.44 log_10_ colony forming units per rod (CFU/rod). Biofilm inocula persisted on positive-control rod explants with ∼6.16 log_10_ CFU/rod. There was ∼6.35 log_10_ CFU/g of tissue in the positive controls versus no identifiable bioburden in the negative controls. Positive controls displayed hallmarks of deep spine infection and osteomyelitis, with robust local tissue response, bone resorption, and demineralization. MB staining was more intense in infected, positive control sites. This work presents an animal-efficient sheep model displaying clinically relevant implant-associated deep spine infection.

## Highlights

• Biofilm inocula reliably produces implant-associated deep spine infection in an ovine model.
• Positive control sites displayed hallmarks of deep spine infection within the 28-day study window.
• Hematogenous spread was not observed from positive to negative controls sites.

## 1 Introduction

Implant-associated spine infection (IASI) is a devastating surgical complication [1]. Effective management of IASI may require multiple surgeries to debride wounds and prolonged culture-specific antibiotics. Ultimately, if eradication of deep infection is unsuccessful, removal of implants may be required, which can lead to deformity, neurologic compromise, disability, and even death [2,3]. Treatment of IASI is fundamentally different than treatment of native or post-operative spine infection without implants due to the presence of hardware which can serve as a nidus for biofilm formation [[4], [5], [6]]. IASI also differs from periprosthetic joint infection (PJI) due to soft tissue milieu and a need for implants to maintain obligate spinal stability [7]. Thus, while IASI poses significant challenges for eradication, many concepts can be applied from the management of PJI where the emphasis is on debridement and implant exchange [[8], [9], [10], [11]].

Understanding how biofilms potentiate infection in the spine space is paramount to successful treatment and eradication of IASI. Biofilm communities provide constituent bacteria substantial protection against antimicrobial agents and the host immune response [12,13]. Specifically, infectious bacteria form matrix-enclosed biofilm communities to evade phagocytic clearance by host neutrophils [14]. A phenotypic subset of biofilm cells is tolerant to antibiotic concentrations many orders of magnitude greater than would otherwise kill planktonic phenotypes [15,16]. Biofilms become a nidus of infection as metabolically quiescent phenotypes outlast antibiotic treatments to subsequently reseed infection once a systemic antibiotic therapy has been removed [[17], [18], [19], [20], [21]]. The need for continual suppressive antibiotics is a hallmark of a biofilm-related infection.

*Staphylococcus aureus* is the leading cause of nosocomial and community acquired infections, it is also the most common organism implicated in IASI [[22], [23], [24]]. Current treatment for implant-associated *S. aureus* infections is predicated on removing the implanted device due to persistence of *S. aureus* biofilms. This is borne out in the PJI literature with high rates of infectious failure following treatment with irrigation and debridement and prosthetic retention versus two-stage revisions [10,11,25]. As implant removal is not always an option in spine surgery, a robust and generalizable large animal model in which to study IASI is greatly needed to develop treatment strategies and technologies to mitigate and eradicate biofilm-related IASIs [26].

Currently, a large animal model of IASI does not exist. While several small animal models of IASI have been described, due to substantial limitations, their results are not broadly extrapolatable to humans [[27], [28], [29], [30], [31], [32], [33], [34]]. Recent studies have highlighted the value of the large animal sheep model for spine applications due to substantial homology to human conditions. The ovine model is particularly appealing due to the ability to place clinically relevant hardware as well as validated use of biofilm as initial inocula for musculoskeletal infection research [5,6,[34], [35], [36], [37], [38]]. Thus, the purpose of the present study was to develop and validate a sheep model for IASI utilizing biofilm as initial inocula as well as use of methylene blue (MB) to visualize and guide debridement of biofilm *in vivo*.

## 2 Materials and methods

### 2.1 Growth of biofilm inocula on spinal fusion hardware

**Fig. 1:**
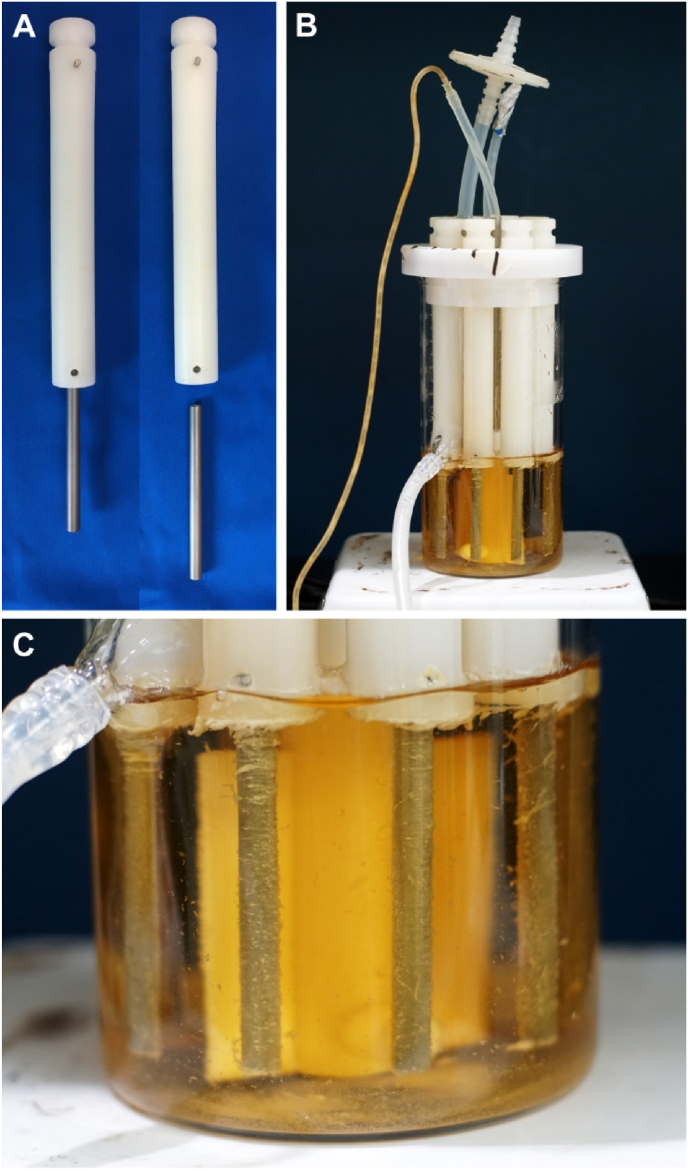
**Reactor system for growing biofilm inocula on spinal fusion rods**. (A) CDC biofilm reactor sample holders modified for spinal fusion rods. (B) The assembled reactor system perfused with fresh broth through the top and waste exiting through the bottom. (C) Close up view of biofilm-covered spinal fusion rods at the end of the 48-h growth period.

Titanium spinal fusion rods were prepared by cutting 5.5 mm diameter rods to standardized 75 mm lengths (DePuy Spine, Raynham, MA, USA; Fig. 1A). Rods were cleaned by sonication in detergent, rinsed with DI-H_2_O, and sterilized by autoclaving in sealed pouches. *Staphylococcus aureus* ATCC 6538 biofilms were grown on the spinal fusion rod sections, for the positive sites of infection, using a CDC biofilm reactor with modified reactor arm rod holders (BioSurface Technologies Corporation, Bozeman, MT, USA; catalog #CBR 2203; Fig. 1A). Reactor arm rod holders were customized by cutting CBR 2203 holders to 150 mm lengths; drilling a 15 mm deep x 5.9 mm diameter hole centered on the bottom of each arm; and installing a M2.5 x 0.45 setscrew 7 mm from the bottom of each arm (Fig. 1A). The assembled reactor systems, loaded with 8 spinal fusion rods each, were sterilized by autoclaving.

Biofilms were grown using established protocols for the CDC reactor system^37,38^. Briefly, each reactor was filled with 500 ml of sterile 100% brain heart infusion (BHI) broth and inoculated with 1 ml of a 0.5 McFarland standard (∼7.5 × 10^7^ colony forming units (CFU)/ml) of *S. aureus* ATCC 6538 prepared from a 24 h tryptic soy broth (TSB) agar culture that was streaked from frozen stock. Each reactor was incubated, in batch phase, on a hotplate set at 34°C for 24 h at 130 RPM baffle rotation. After the batch phase, each reactor was perfused with 10% BHI at 6.4 ml/min for an additional 24 h. Quantification of biofilm bioburden was performed on a representative rod from each reactor by dispersing the respective biofilm into 18 mL of fresh 1x phosphate buffered saline (PBS) mirroring established protocols: sequentially vortexing for 1 min, sonicating for 10 min at 42 kHz, and vortexing for 10 s. A log_10_ serial dilution of the suspended bacteria in the 1x PBS was performed and plated on TSB agar using established procedures. Plates were incubated for 24 h at 37 °C after which colonies were counted.

Rods containing biofilms intended for inoculation were transported to the operating room individually in 50 ml of fresh 10% BHI and used within 2 h of removal from the biofilm reactor.

### 2.2 Surgical approach

All animal work was performed with approval from the Institutional Animal Care and Use Committee (IACUC Approval #19–05008) and the Environmental Health and Safety (EHS) office of the University of Utah. For this study, female Rambouillet sheep (n = 5) between 2 and 3 years age and weighing 90 ± 20 kg (K Bar Live-stock, Manta, TX, USA) were used. The animals are labeled numbers 1–5 herein. A fellowship-trained spine surgeon performed all surgical procedures. Prior to surgery, the sheep were fasted for >12 h. Sheep were initially anesthetized using an intravenous (IV) injection of Propofol (5–10 mg/kg) to allow for endotracheal tube intubation; the sheep were then placed in the sternal recumbency position and maintained under anesthesia with isoflurane (ranged from ∼0.5 to 5%). An IV catheter was placed in the left forelimb and 0.9% saline was administered at a rate of 10 ml/kg/hr. Veterinary technicians monitored the sheep’s heart rate, temperature, carbon dioxide and oxygen levels, and respiration throughout the survival and terminal surgical procedures.

In a first surgical procedure, the animals received perioperative 2 mg/kg IM dose of ceftiofur to prevent postoperative systemic sepsis [39]. The dorsal thoracic and lumbar spine was prepped by shaving the wool, and draping in the normal sterile fashion with sterile povidone iodine prep. Two 15 cm incisions were marked out and separated by a skin bridge of 15 cm. The extent of the cranial incision was at the level of the superior border of the scapula, subsequent incisions were made caudal to this mark. Each animal had both positive and negative control sites of infection, randomized with regard to thoracic vs lumbar and left vs right placement. Beginning with the negative control site (sterile), whether that be in the thoracic (T3-T7) or lumbar spine (L1-L4), a posterior mini-open approach to the spine was performed. Fascia was incised unilaterally and dissection was completed, exposing the lamina and facets in the normal fashion. Using local landmarks, pedicle screws were placed in free-hand fashion [40]. Standardized 6.5 mm × 35 mm pedicle screws were used throughout (Stryker Spine, Kalamazoo, MI, USA). With the pedicle screw in place the sterile 75 mm titanium alloy 5.5 mm rod was affixed to the pedicle screw tulip. The wound was then irrigated with 500 cc of normal saline. The incision was closed in a layered fashion, beginning with a running 0-PDS for the fascia, followed by running 2-0 PDS for the subcutaneous tissue and finally a running 2-0 Nylon for the skin. The skin was glued and an occlusive dressing placed.

Once the sterile wound had been sealed, the same procedure was repeated for the designated positive control site (inoculated), however, with unilateral dissection occurring on the contralateral side of the spine. After the pedicle screw was secured, the *S. aureus* biofilm-covered rod was aseptically removed from the 50 ml BHI broth using sterile forceps and affixed to the pedicle screw tulip. A layered closure was performed as outlined above. Fluoroscopy was used to confirm hardware position.

For post-operative analgesia, each sheep was given two fentanyl patches (100 μg/kg/hr), placed on a shaved area on the forelimb and secured with vet wrap and tape as necessary. Post anesthesia monitoring extended until the animals could stand on their own, eat, and drink. No further ceftiorfur or other antibiotics were administered.

Throughout the course of the study, research and veterinary teams monitored each sheep daily to assess symptoms of pain and distress. Sheep were monitored for the duration of the 28-day study.

Prior to euthanasia on Day 28, a terminal surgical procedure was performed. The animals were returned to the operating room following the same methods as outlined above: each was again anesthetized, the skin was prepared, and the site draped as above. The negative control wound was surgically interrogated first. The incision site and underlying fascia were reopened using meticulous sterile technique. Each surgical site was sampled with a commercially available culture swab using the Z-technique: rotating the swab as the tip is zigzagged across the wound while avoiding the surgical wound edge [41]. Digital photographs (using a Nikon D7000 camera) of the wound were obtained under controlled ambient lighting. Once the implant was exposed, the wound was photographed and then stained for 5 min with 200 cc of 0.05% MB solution comprised of ProvayBlue™ aseptically diluted in sterile 0.9% saline [42]. The wound was then irrigated in triplicate with normal saline and photographed again with the same exposure settings and camera position as the pre-stain photograph.

Approximately 1 g tissue samples were obtained in triplicate from representative areas. The locations of these tissue samples were marked in the digital photographs for reference with MB stain intensity.

These same procedures were performed on the positive control infection site. After sites were surgically explored, the rods were removed and either immediately fixed in modified Karnovsky’s fixative for analysis by scanning election microscopy (sheep numbers 1–2), or transported back to the laboratory in 18 mL of sterile PBS and used for the microbiological bioburden analysis (sheep number 3–5). At the completion of sample acquisition, the sheep was euthanized via Beuthanasia solution (1 mL/4.5 kg). Post-mortem necropsy was then performed on the thoracic and lumbar vertebrae, with implanted pedicle screws still intact. Vertebral specimens were harvested and fixed in 10% neutral buffered formalin (NBF). The vertebrae were submitted for micro-computed tomography (micro-CT) and non-decalcified histological analyses.

### 2.3 Microbiology

Quantification of rod bioburden was performed by first dispersing surface-associated bioburden into 18 mL of 1x phosphate buffered saline (PBS) by sequentially vortexing for 1 min, sonicating for 10 min at 42 kHz and vortexing for 10 s. A ten-fold serial dilution plating of the suspended bacteria in the 1x PBS was performed and plated on Columbia blood agar. The bacterial bioburden of site tissue samples was quantified using an established procedure^41^. Each sample was first weighed, then homogenized in 2 ml of sterile PBS for 2 min with a disposable tissue grinder. A ten-fold serial dilution plating of the homogenized tissue suspension was performed in PBS and plated on Columbia blood agar. Plates were incubated for 24 h at 37 °C after which colonies were counted. Surgical site swabs were cultured using a streak plate method on Columbia blood agar and incubated for 24 h at 37 °C.

Scanning election Microscopy (SEM) of Implants:

Spinal fusion rod explants and controls from the biofilm reactors were fixed in modified Karnovsky’s fixative (2 h), dehydrated in ascending concentrations of 70%, 95%, and 100% ethanol (1 h each), air dried, gold sputter-coated in a Hummer 6.2 sputter coater (Anatech, Battle Creek, MI), and imaged in a JEOL JSM-6100 tungsten filament scanning electron microscope ((JEOL, Peabody, MA).

### 2.4 Radiography and micro-computed tomography

The vertebrae, with intact pedicle screws, were individually radiographed in the transverse plane with a cabinet x-ray system (Faxitron 43855A, Wheeling, IL) set to 90 kV for 3 min. Following specimen fixation, the thoracic specimens (Control + Infected) were prepared for micro-CT imaging. The excess soft tissue was dissected and the pedicle screws were carefully removed without disturbing the bone. The screws were removed in all thoracic specimens to prevent metal artifacts. The dissected thoracic specimens were scanned using a Quantum GX micro-CT (PerkinElmer; Waltham, MA.) with a tube voltage of 90 kVp and tube current of 200 μA^42^. The field-of-view (FOV) varied depending on the region of interest (ROI). The body of the vertebrae were scanned at a 73 mm field-of-view (FOV); the spinous process was scanned at a 30 mm FOV to increase the resolution of the bones outer surface where the most significant bone response occurs. Micro-CT scans were used to assess signs of osteomyelitis from infection and/or possible disturbance from the surgical procedure. The scans were imported into FIJI (Image J) and MountainsMap® 7 (Digital Surf, Besançon, France) analysis software, which allowed for both qualitative cross-sectional and 3D rendered analyses.

### 2.5 Histological processing and imagining

For specimens undergoing histologic analysis, the hardware remained in the vertebral body throughout processing. To preserve, chemically fix, and embed the bone in these specimens, excess soft tissue was removed as well as a small portion of the ventral region of the vertebral body to allow for adequate infiltration through the cortical shell into the dense cancellous bone at the screw/host bone interface. The spinous and transverse processes remained unaltered and intact through the entire process. Using established protocols, the specimens were dehydrated in a graded series of ethanol, infiltrated, and embedded in poly-methyl-methacrylate (PMMA) allowing for undecalcified histological analyses without the need to remove the pedicle screw [[43], [44], [45]]. Longitudinal sections were captured directly through the pedicle screw using a variable-speed grinding wheel (Buehler Incorporated, Lake Bluff, IL). Once the screw threads were grossly visible, the specimens were polished to an optical finish and coated with a thin conductive layer of carbon for ∼30s with a High Vacuum Turbo Carbon Coater 208C (Ted Pella, Redding, CA) allowing for backscattered electron (BSE) imaging using a JEOL JSM-6610 (JEOL, Peabody, MA) scanning electron microscope (SEM) [46]. Six digital images were captured at 30x magnification with a resolution of 2560 × 1920 pixels across the entire vertebra. Post image processing was performed using Microsoft Research Image Composite Editor (MRICE) to mosaic/stitch the BSE images together creating a high-resolution overhead view of the screw/vertebra. After BSE imaging, the thin carbon coating was removed and the specimens were adhered to individual plastic slides using Exakt Technovit 7210 and a precision adhesive press (EXAKT Technologies, Oklahoma City, OK) and ground to a final thickness of ∼100 μm. The sections were stained with Sanderson’s Rapid Bone Stain (SRBS) and viewed under a light microscope (Nikon E600, Nikon Inc., Melville, NY) [47,48].

### 2.6 Photographic analysis of MB-stained sites

Images from the surgical sites were analyzed and compared with the microbiological data from selected sites. Image analysis was performed to quantify blue tissue staining using red channel intensity using ImageJ software (National Institutes of Health, Bethesda MD) according to described techniques [42]. For each surgical site, four distinct regions were analyzed for both the pre- and post-MB stained fixed-exposure photographs: a 1 cm diameter circle around each of the three biopsy sites, and all the exposed tissues of the surgical site. The extent of MB staining was determined by percent change in red channel intensity, for each of the 4 regions analyzed from images pre- and post-MB staining images.

## 3 Results

### 3.1 Biofilm inoculum

**Fig. 2:**
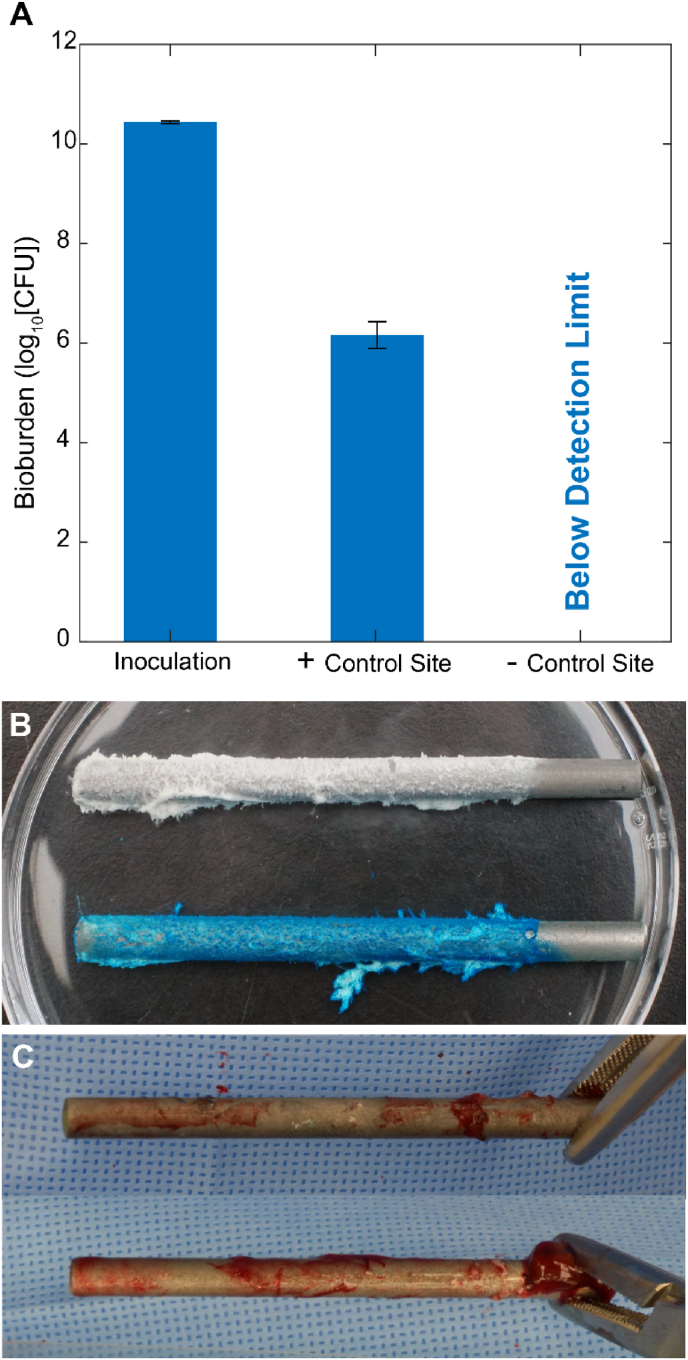
**Bacterial bioburden and MB staining of representative spinal fusion rods.** (A) Average bacterial bioburden on representative rods used for inoculation (left), the explant rods removed from the positive (middle) and negative (right) infection control sites of sheep #3–5 (error bars are ± S.D.). (B) Close up photograph of representative inoculation rods without (top) and with (bottom) 5 min staining in 0.05% MB. (C) Representative explant rods from negative (top) and positive (bottom) infection control sites after staining in 0.05% MB.

**Fig. 3:**
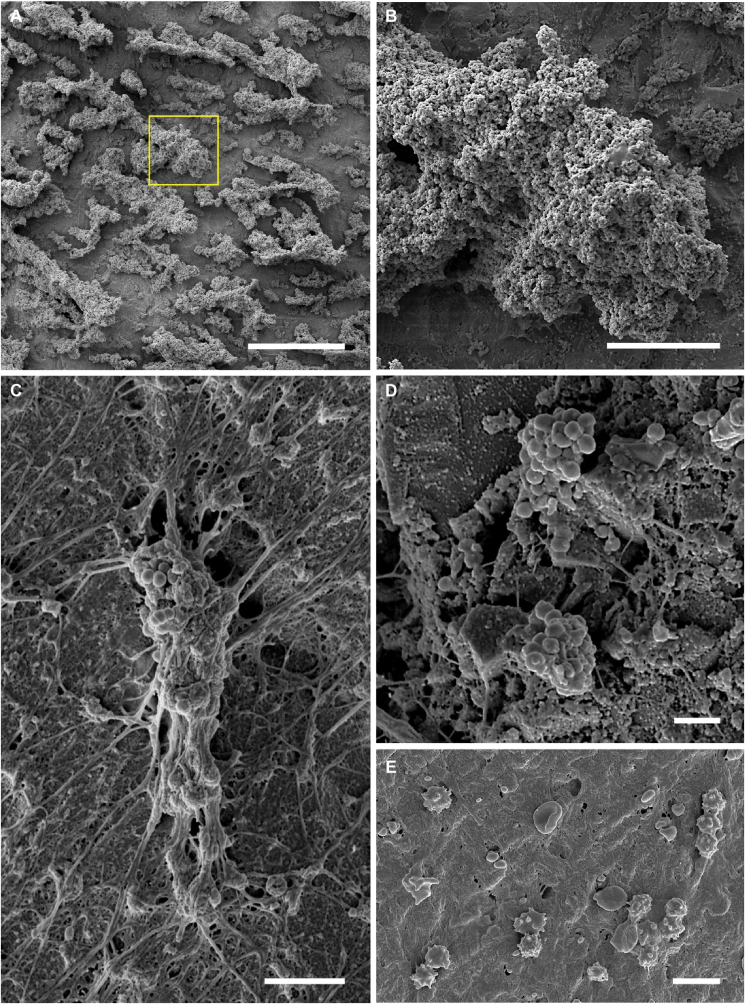
**Scanning electron micrographs of representative spinal fusion rods.** (A–B) Surface of the rods used for inoculation showed large plumes of biofilm. Scale bars are 250 and 5 μm, respectively. The yellow box in panel A is the location of panel B. (C–D) Representative explant rods from positive infection control sites displayed small aggregates of bacterial biofilm with extensive fibrous host response and encapsulation. Scale bars are 5 and 2 μm, respectively. (E) Representative explant rods from negative infection control sites showed fibrous host response and macrophage multinucleation but no evidence of bacterial bioburden. Scale bar is 10 μm. (For interpretation of the references to colour in this figure legend, the reader is referred to the Web version of this article.)

**Fig. 4:**
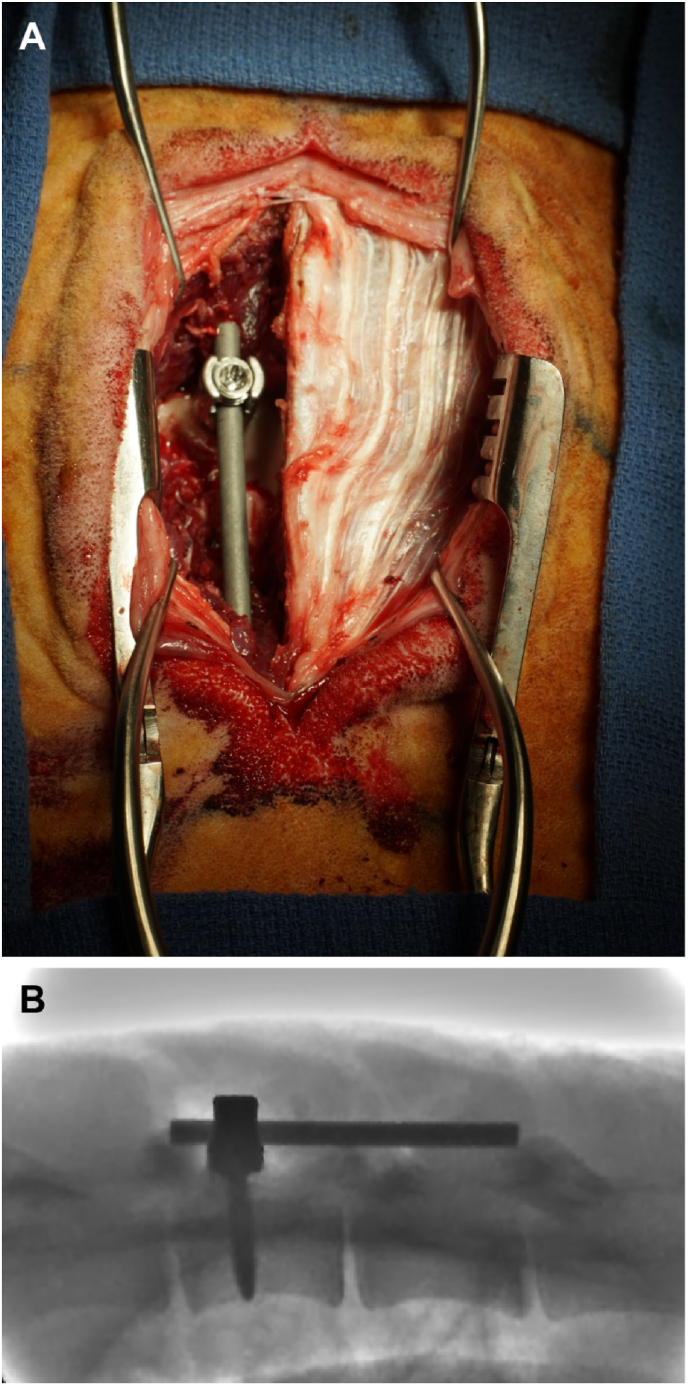
**Representative placement of spinal fusion rod section in survival surgery.** (A) Photograph of the surgical site just before closure. (B) Lateral fluoroscopy image of the of the closed surgical site to visualize the placement of fusion hardware in this animal model.

The biofilm inocula on representative rods from each modified CDC biofilm reactor run (Fig. 1) ranged narrowly from 10.41 to 10.45 log_10_ CFU corresponding with a bioburden surface density from 9.28 to 9.33 log_10_ CFU/cm^2^ (Fig. 2A). Biofilms on the reactor control rods were visible to the naked eye (Fig. 1, Fig. 2B) and were avidly blue after 5 min of 0.05% MB staining (Fig. 2B). These biofilms were observed as large sheets and plumes in scanning electron micrographs (Fig. 3A & B). Fig. 4 visually and radiographically shows surgical rod placement for both positive and negative infection control sites.

### 3.2 Surgical follow up

All 5 sheep awoke from anesthesia without complications and were able to stand within 2 h of the operation. During daily monitoring of the sheep, no animals displayed signs of systemic infection or sepsis. Diets and activity were maintained for each animal throughout the follow up period. No animal required further local wound care or antibiotic due to systemic illness. All animals survived until the 28-day study endpoint.

### 3.3 Infection outcomes and microbiology

**Fig. 5:**
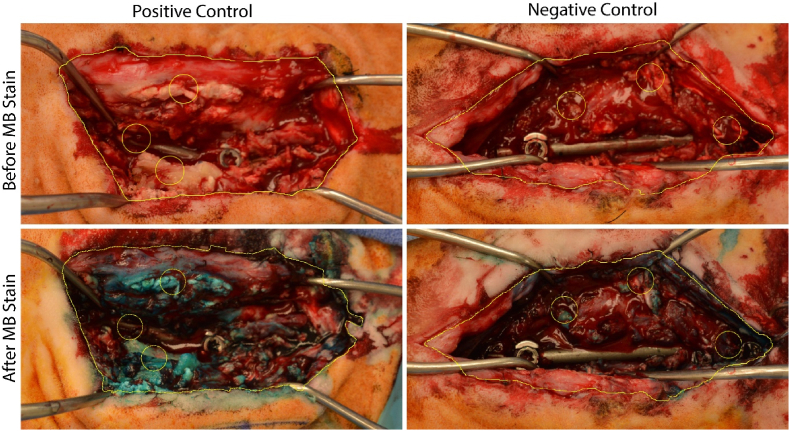
**Representative photographs of surgical sites at the terminal procedure 28-day endpoint.** (Top row) Positive and Negative controls of infection after surgically opening the sites. (Bottom row) The respective surgical sites after staining with 0.05% MB for 5 min. The 1 cm yellow circles mark both the locations where tissues were biopsied for microbiology and the visual regions of the photographs used for blueness analysis by changes in red-channel intensity. The yellow manual trace around the surgical incision was also used in blueness analysis by changes in red-channel intensity of the whole respective surgical site. (For interpretation of the references to colour in this figure legend, the reader is referred to the Web version of this article.)

**Fig. 6:**
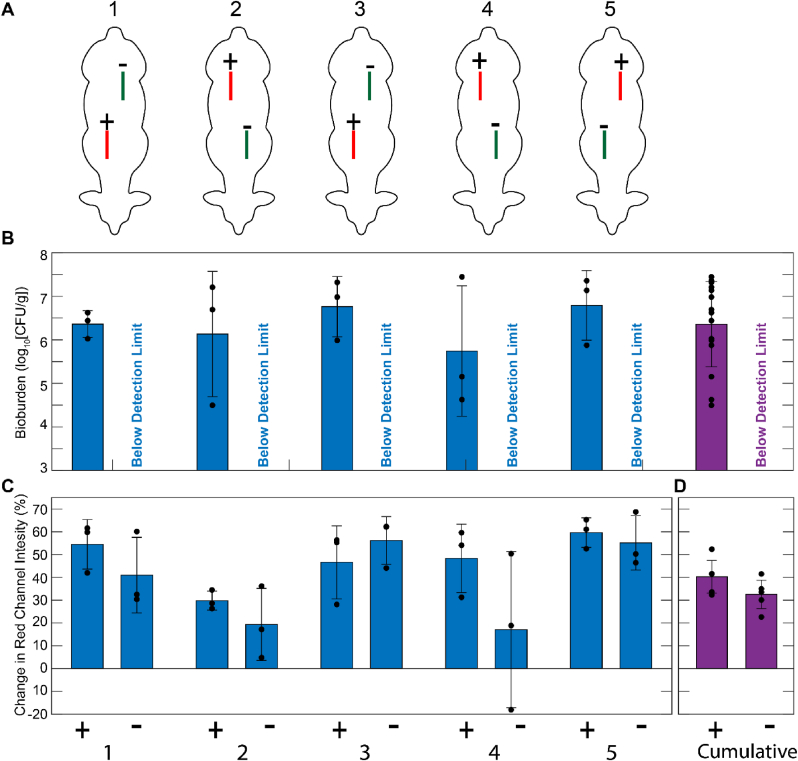
**Quantitative outputs from microbiology and MB staining analyses.** (A) The randomized placement of the negative (Green) and positive (Red) control sites in the 5 respective study animals. (B) The average bioburden in the three biopsies from each surgical site (see Fig. 5) is given by the blue bars. The aggregate average bioburdens for the positive and negative control sites are given by the purple bars. (C) The average percent change in red channel intensity is given by the blue bars for the 1 cm diameter circle around each of the three biopsy locations in each surgical site (see Fig. 5). (D) The average percent change in red channel intensity is given by the purple bars for the zone within the surgical access incision for the five negative and five positive control sites. Black circular markers are individual data points and error bars are ± S.D. (For interpretation of the references to colour in this figure legend, the reader is referred to the Web version of this article.)

Across all five animals, infected positive control sites visually displayed hallmark features of infection with redness, purulence, necrotic tissue (Fig. 5) and notable loosening of the pedicle screws. Similarly, every culture swab taken from a positive infection control site produced a positive culture for *S. aureus.* The tissue biopsies in the positive control sites had an average bioburden of 6.36 ± 0.98 log_10_ CFU/g ranging from 4.50 to 7.44 log_10_ CFU/g (Fig. 6B). In every positive control site, there was *S. aureus* tissue bioburden exceeding the 10^5^ CFU/g threshold quantitatively defining infection^48^. The average bioburden on rods retrieved from positive control sites was 6.16 log_10_ CFU/rod ranging from 5.86 to 6.34 log_10_ CFU/rod corresponding with a bioburden surface density from 4.73 to 5.22 log_10_ CFU/cm^2^ (Fig. 2A). Notably, the prophylactic ceftiofur antibiotic course did not eliminate the biofilms in inoculated sites and did not prevent bacteria from infecting soft tissue (Fig. 2, Fig. 6B).

On scanning electron micrographs cocci were visible on explant rods as aggregates associated with extensive host fibrous tissue (Fig. 3C & D). In contrast, for all negative control sites, there was no visible evidence of infection or evidence of pedicle screw loosening. In negative controls, bacterial bioburden was below detectable thresholds for all tissues and hardware specimens (Fig. 2A). Every culture swab taken from the negative infection control sites produced negative cultures; not a single organism was visually identifiable on the culture plates after a 24 h incubation. Likewise, bacterial cocci were not visible in scanning electron micrographs of rod explants; negative site rod explants were encapsulated with fibrous tissue and showed a comparatively high number of associated macrophages in various stages of multinucleation (Fig. 3E).

### 3.4 Radiography and computed tomography

**Fig. 7:**
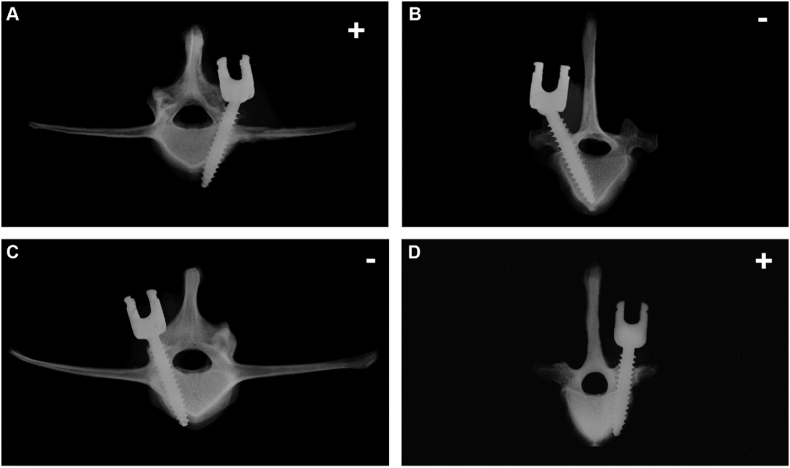
**Representative cabinet x-rays of dissected vertebrae in the transverse plane**. Positive control sites included are one lumbar (A) and one thoracic (D) vertebrae. Negative control sites included are one lumbar (C) and one thoracic (B) vertebrae. Positive control sites are indicated by + and negative control sites by -.

Post-necropsy radiographs of vertebrae demonstrated appropriate placement of screws through the pedicle and into the anterior vertebral body as displayed by representative specimens in Fig. 7. There was bicortical fixation noted in several specimens due to standardized minimum available implant length (35 mm). The vertebrae of positive control sites showed radiographic evidence of periosteal reaction, bone loss and/or cortical irregularities in the spinous and/or transverse process to a greater extent than the vertebrae of negative control sites (Fig. 7).

**Fig. 8:**
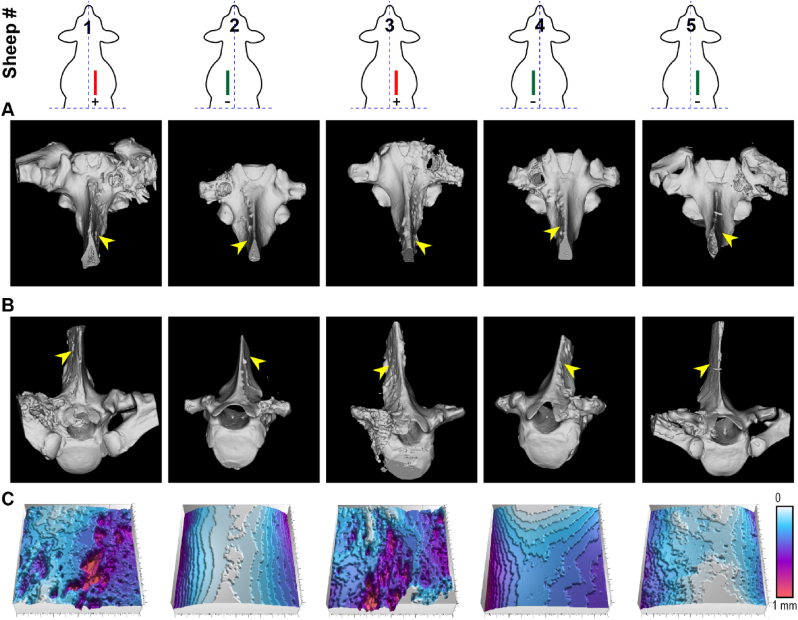
**Micro-CT analysis of all thoracic vertebrae.** (Top) The randomized surgical site locations of the negative (Green) and positive (Red) control sites in the five respective thoracic sheep sites comprising three negative and two positive controls. (A) Dorsal-ventral and (B) anterior-posterior view of the vertebrae. (C) Surface topographies from the side of the spinous processes indicated with the yellow arrows from panel A & B. (For interpretation of the references to colour in this figure legend, the reader is referred to the Web version of this article.)

Micro-CT imaging was obtained at the level of the pedicle screw with the pedicle screw transected along its longitudinal axis (Fig. 8A & B). The cross-sectional and 3D-rendered micro-CT scans of the infected specimens further revealed significant pockets of bone resorption within the spinous process in the regions adjacent to the head/rod of the pedicle screw; cortical bone deterioration was evident at the intersection of the tulip and screw threads (Fig. 8). From these sections a significant cortical bone response and an endosteal response indicative of new bone formation could be seen in positive control sites. There was also cortical bone deterioration along both the cranial and caudal portions of the transverse and spinous processes. This was in contrast to the negative control vertebrae, which demonstrated negligible signs of bone resorption; the negative control sites showed minimal cortical periosteal scalloping or bone loss surrounding the screw threads (Fig. 8).

### 3.5 Histology

**Fig. 9:**
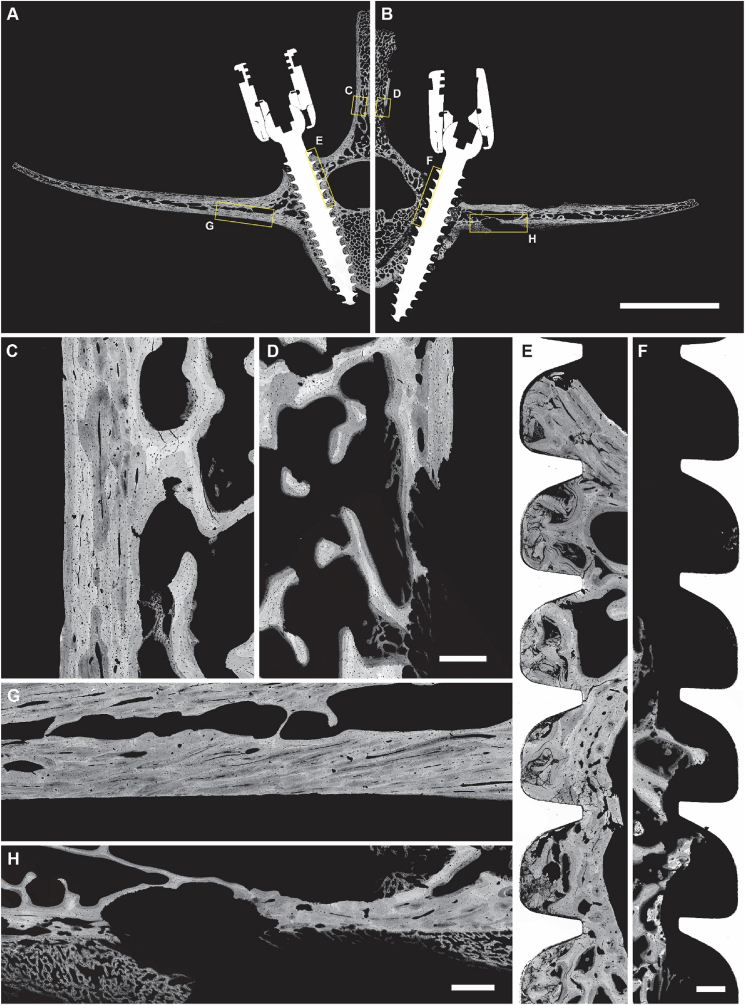
**SEM BSE image mosaics of hard-plastic embedded lumbar sites with intact pedicle screws.** Low-magnification view of the negative (A) and positive (B) sites of infection. Scale bar is 20 mm. Yellow boxes indicate the regions of higher resolution panels C–F. (C, D) The edge of the spinous process (scale bar is 500 μm), (E, F) the medial portion of the pedicle screw-bone interface (scale bar is 500 μm), (G, H) and the ventral side of the transverse process for negative and positive control sites, respectively (scale bar is 1000 μm).

**Fig. 10:**
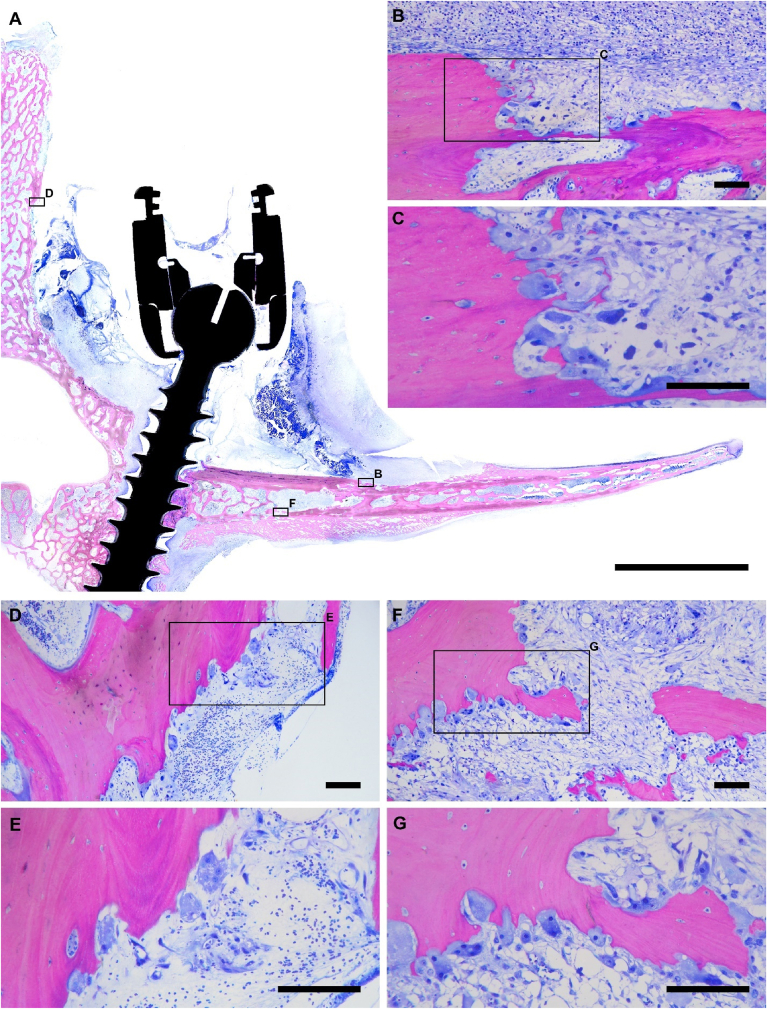
**Light microscopy image mosaics of hard-plastic embedded lumbar site with intact pedicle screw stained with SRBS.** (A) Low-magnification view of the positive control site of infection. Black boxes indicate the regions of higher resolution (panels B–G). Scale bar is 10 mm. High resolution insets showing bone resorption and osteoclast activity on the dorsal side of the transverse process (B, C), the lateral face of the spinous process (D, E), and the ventral face of the transverse process (F, G). Scale bars are 100 μm for panels B–G.

Qualitatively, the BSE and light histological analyses were consistent with radiographic findings demonstrating bone resorption in infected specimens (Fig. 9, Fig. 10); specifically, the higher resolution BSE images revealed significant resorption along the spinous and transverse processes adjacent to the pedicle screws (Fig. 9). In addition to resorption, infected bone specimens both showed significant periosteal reaction (Fig. 9H). Positive control specimens showed signs of regional cancellous bone resorption around the pedicle screws consistent with micromotion at the host/implant interface.

In contrast, negative control vertebrae lacked significant periosteal reaction and displayed minimal signs of resorption (Fig. 9A, C, E, G). The host cancellous bone adjacent to screw threads was in a quiescent state with no signs of bone consolidation, consistent with limited time *in situ* (Fig. 9E). Similarly, there was no histologic evidence of implant loosening (Fig. 10). The histological analysis by light microscopy coincided with BSE analysis demonstrating a high activity of osteoclasts in the positive control lumbar vertebrae, as well as neutrophilic infiltrate and soft tissue reaction (Fig. 10). The extent of bone resorption varied from vertebra to vertebra, all positive control vertebrae showed bone resorption exceeding that observed in negative control vertebrae (Fig. 9, Fig. 10).

### 3.6 Surgical site MB staining

MB staining was more intense in positive control sites compared to negative control sites (Fig. 5). The average red channel intensity at tissue biopsy sites was higher in positive control sites (p = 0.088, Fig. 6C). The average red channel intensity for the entire surgical site was higher for positive control sites of infection vs negative control sites (p = 0.071, Fig. 6D).

## 4 Discussion

Because of the persistent nature of IASI, a large animal model of IASI is needed to study this challenging entity and develop treatment strategies to mitigate and eradicate biofilm-related IASI [4]. To that end the present study presents the development and validation of a large animal sheep model for IASI utilizing *S. aureus* biofilm as initial inocula as well as use of MB to visualize biofilm and/or devitalized tissues *in vivo*.

Importantly, all animals survived until the natural 28-day study endpoint, all positive control sites developed infection, and none of the negative control sites showed any signs of infection through hematogenous spread or surgical contamination. Other large animal models wherein planktonic bacteria are used as initial inocula can lead to excessively virulent infections, diminished animal welfare, and frequent early termination [49]. Moreover, many planktonic-inocula models produce inconsistent infection outcomes with a considerable percentage of target sites which do not develop the intended infection [[50], [51], [52], [53], [54], [55], [56], [57]]. Use of biofilm as inocula allowed reliable induction of deep infection in all target sites without systemic sepsis or bacteremia yet did not affect the overall well-being of the animals within the study window. The use of positive and negative control sites within a single animal allowed increased animal efficiency and enhanced statistical power. While novel in a large animal model, there is precedent for use of both positive and negative controls in the same animal [28]. Surgical techniques used best practices and mirrored those found in human spine surgery. Use of clinically relevant pedicle screw and rod instrumentation allows for easy translation to human spine conditions.

Multiple radiographic approaches including intraoperative fluoroscopy, and post-mortem x-ray and micro-CT enabled detailed evaluation of both implant position as well as bony hallmarks of infection. Due to standardized implant length, there was bicortical fixation on several specimens, however, all implants were in acceptable position. Bicortical fixation in this setting was of no clinical consequence and there was no clinically significant medial screw breach [58,59]. Radiographic analysis demonstrated that all infected vertebrae had evidence of periosteal reaction, bone loss and/or cortical irregularities in the spinous and/or transverse process consistent with osteomyelitis [38,57,[60], [61], [62]]. Positive control specimens also showed signs of regional cancellous bone resorption around the pedicle screws consistent with micromotion at the host-implant interface and this mirrored clinical findings of loose hardware in infected surgical sites [63]. These radiographic findings and presence of screw loosening in the setting of known infection are of clinical relevance, as these commonly occur in human patients with IASI [64]. The successful reproduction of this clinical paradigm serves to further validate the fidelity of the model for future studies and analyses.

MB staining was observed to be more intense in positive controls when compared to negative control wounds, this finding was visually apparent and trended towards significance (p = 0.07, Fig. 6D). More intense MB staining was noted specifically in areas with higher concentrations of infectious material and eschar from electrocautery. Infected and non-viable tissue have previously been found to stain preferentially with MB in existing *in vitro* and *in vivo* studies [9,42]. The differential staining effect between infected and sterile wounds was likely confounded by staining of eschar from electrocautery which was present in both infected and sterile wounds. This effect could be minimized in future studies by avoiding use of electrocautery. Additionally, limitations intrinsic to the use of live tissue such as pooling blood and lighting a multiplanar wound could be minimized by completing all MB staining and imaging post-mortem. Post-mortem study, however, would limit comparison to human surgery which is performed in live patients with perfused tissues. In aggregate, use of electrocautery and live tissue likely account for smaller than anticipated differences in staining seen between infected and sterile wounds based on prior *in vitro* works [42]. Nonetheless, despite these limitations and small sample size due to the pilot nature of this study, differential MB staining trended towards significance when staining biofilm and nonviable tissue *in vivo.* These findings, suggest that MB may be a valuable debridement aid for surgeons to identify and remove infected and devitalized tissues. A larger study is warranted to more completely describe MB staining *in vivo.*

Prior to the present study, a large animal model of IASI did not exist [4]. Several small animal models of IASI have been developed over the last two decades in rabbits, rats and mice. These animals, however, frequently resolve implant and biofilm-associated infections without additional intervention, outcomes which are fundamentally contrasted with those of a human host [4,17,19,[27], [28], [29],^31^,^63^,^65^]. Large animal models in both canines and sheep have been developed for osteomyelitis and diskitis but have not, to date, been used for implant-associated spine infections [[4], [5], [6],^33^,^35^,^66^,^67^]. Larger animals are appealing for use in implant-associated infection models as they allow for the use and study of clinically relevant implants. Use of canines for large animal studies, in particular, is problematic as they are commonly kept as pets in large portions of the globe making sheep models more appealing [4]. Additionally, a growing body of literature has supported the use of sheep for a validated open tibia fracture model which uniquely uses biofilm as initial inocula; this model was, in part, inspirational for the development of the present study [38].

The present study used biofilm phenotypes as inocula rather than planktonic liquid-borne inocula. Historically, laboratory techniques have relied on the use of log-phase-replicating planktonic cultures. These entrenched microbiology practices were conscripted for use in the first experimental infection models and have since become commonplace. Yet, as a whole, it is estimated that over 99% of opportunistic bacterial pathogens reside in stationary phenotypes, including the resident skin flora commonly implicated as culprits in surgical site infections [68,69]. Planktonic cells are more readily cleared by the immune system, potentially explaining the typically high number of positive controls which do not develop infection with planktonic inocula; reported infection rates with planktonic inocula in literature references ranged broadly from 47 to 94% [15,[51], [52], [53], [54], [55], [56],[70], [71], [72]].

Moreover, planktonic bacteria are much more susceptible to antibiotic interventions and do not immediately display the desired recalcitrance observed in the worst implant associated clinical infections [12]. Because planktonic bacteria produce acute infections which are less chronic in nature; this in turn frequently causes intense infection signals, septicemia, bacteremia, and in some cases an untimely loss of the study animal [49]. Finally, there are logistical limitations when using planktonic bacteria in the dynamic weeping and bleeding surgical site tissues. Liquid inocula do not easily stay put, producing variability in the amount and location of inocula. Inoculating with biofilms, as shown here, reliably produces the hallmarks of deep tissue infection without the undesired acute components of a planktonic infection; this mirrors the challenges observed clinically accompanying spinal fusion procedures. As animals fared well during the monitoring period, yet had known biofilms dwelling in positive control sites, it prompted a clinical consideration: are biofilm-related spine infections misdiagnosed (perhaps early on) as surgery-related pain? We anticipate future work being performed to explore this consideration.

Since the mid-1900’s, infection has been described quantitatively as a bioburden exceeding 10^5^ CFU per gram of tissue or ml of body fluid [73]. The current model exceeds this threshold, with positive control site tissues having bioburdens greater than 10^6^ CFU per gram.

Despite this clear laboratory definition, diagnosing IASI in clinical practice is nuanced and there is no clinically useful consensus agreement defining IASI as has been done for PJI [8,74,75]. Yet guidelines from consensus definitions of PJI can be generalized to IASI. To that end, the current model reliably establishes deep spine infection and thus provides an opportunity to refine the definition of IASI by validating proposed diagnostic criteria.

The present study had several limitations. The results are based on the use of a single species of microorganism, while *S. aureus* is both the most common cause of significant postoperative spine infection and considered the prototypical Gram-positive agent for infectious models, its growth patterns may not be extrapolatable to all clinically relevant microorganisms [22,23,76]. Lastly, although the model was designed to closely mimic human spinal surgery and subsequent postoperative infection the exact generalizability from a sheep to a human spine model is incompletely understood. However, existing sheep spine models and the open fracture data strongly suggest that there is a high degree of homology between human and sheep models [4,6,35,77].

## 5 Conclusion

The present study developed an animal-efficient sheep model for deep implant-associated spine infection. Use of a single animal capable of harboring both infected and non-infected spine wounds reduced the number of animals needed and increased statistical power. This study shows that *S. aureus* biofilm inocula readily produce clinically relevant deep spine infection that can be visualized with conventional microbiology techniques, radiographic and histologic analyses, as well as electron microscopy and MB staining. This robust and clinically relevant model is foundational for future investigations into novel treatments and therapeutic modalities for implant-associated spine infection.

## Funding

10.13039/501100002732AOSpine North America Young Investigator Research Grant

## Declaration of competing interests

The authors declare the following financial interests/personal relationships which may be considered as potential competing interests:Jeremy D. Shaw reports financial support was provided by 10.13039/501100002732AOSpine North America. Jeremy D. Shaw has patent #Bacterial biofilm staining device and methods of use (#US17/328,578) pending to The University of Utah.

## CRediT authorship contribution statement

**Jeremy D. Shaw:** Conceptualization, Methodology, Investigation, Conducting, Resources, Writing – original draft, Writing – review & editing, Supervision, Project administration, Funding acquisition. **Travis L. Bailey:** Methodology, Investigation, Conducting, Writing – review & editing. **Jemi Ong:** Methodology, Investigation, Data curation, Writing – review & editing. **Darrel S. Brodke:** Conceptualization, Writing – review & editing. **Dustin L. Williams:** Conceptualization, Writing – review & editing, Formal analysis, Funding acquisition. **Richard A. Wawrose:** Writing – review & editing. **Richard T. Epperson:** Writing – review & editing, Data curation, Formal analysis. **Brooke Kawaguchi:** Writing – review & editing, Data curation. **Nicholas N. Ashton:** Methodology, Investigation, Data curation, Formal analysis, Conducting, Writing – original draft, Writing – review & editing, Visualization, Supervision, Project administration.

## Data Availability

Data will be made available on request.
